# Comparative proteomic analysis of *Methanothermobacter thermautotrophicus* reveals methane formation from H_2_ and CO_2_ under different temperature conditions

**DOI:** 10.1002/mbo3.715

**Published:** 2018-09-10

**Authors:** Cong Liu, Lihui Mao, Xiongmin Zheng, Jiangan Yuan, Beijuan Hu, Yaohui Cai, Hongwei Xie, Xiaojue Peng, Xia Ding

**Affiliations:** ^1^ School of Life Sciences and Institute of Life Science Nanchang University Nanchang Jiangxi China; ^2^ Jiangxi Super‐rice Research and Development Center Jiangxi Academy of Agricultural Sciences Nanchang Jiangxi China; ^3^ Biology Experimental Teaching Demonstration Nanchang University Nanchang Jiangxi China

**Keywords:** iTRAQ, methane formation, *Methanothermobacter thermautotrophicus*, proteomics, temperature stress

## Abstract

The growth of all methanogens is limited to a specific temperature range. However, *Methanothermobacter thermautotrophicus* can be found in a variety of natural and artificial environments, the temperatures of which sometimes even exceed the temperature growth ranges of thermophiles. As a result, the extent to which methane production and survival are affected by temperature remains unclear. To investigate the mechanisms of methanogenesis that *Archaea* have evolved to cope with drastic temperature shifts, the responses of *Methanothermobacter thermautotrophicus* to temperature were investigated under a high temperature growth (71°C) and cold shock (4°C) using Isobaric tags for relative and absolute quantitation (iTRAQ). The results showed that methane formation is decreased and that protein folding and degradation are increased in both high‐ and low‐temperature treatments. In addition, proteins predicted to be involved in processing environmental information processing and in cell membrane/wall/envelope biogenesis may play key roles in affecting methane formation and enhancing the response of *M. thermautotrophicus* to temperature stress. Analysis of the genomic locations of the genes corresponding to these temperature‐dependent proteins predicted that 77 of the genes likely to form 32 gene clusters. Here, we assess the response of *M. thermautotrophicus* to different temperatures and provide a new level of understanding of methane formation and cellular putative adaptive responses.

## 1 INTRODUCTION

Methanogens are the key microorganisms responsible for the terminal step of anoxic degradation of organic materials and play a major role in the global carbon cycle in the natural environment. Methane production is the energy‐yielding metabolism of methanogens and methane is a major catabolite unique to these *Archaea* (Ravichandran, Munisamy, Varadharaju, & Natarajan, 2015). There is a growing interest in converting organic wastes into bioenergy sources via methane fermentation in anaerobic digesters. Methanogenesis occurs in a variety of natural and artificial environments, such as anaerobic bioreactors, rice paddy soils, deep subsurface marine environments, gastrointestinal tracts of animals, oil wells, freshwater sediments, and extreme environments (Hendrickson et al., 2008; Li et al., 2015). To understand and predict the production of CH_4_, numerous studies have been conducted. Methane formation is often rate‐limiting and highly sensitive to changes in environmental factors such as temperature. However, methanogens can exhibit a diverse range of temperature adaptation. These environments can range from geothermal vents at temperatures greater than 100°C to oceans at temperatures below 4°C (Reid et al., 2006; Zinder, Anguish, & Cardwell, 1984). Methanogens are common in petroleum reservoirs with temperatures in the range from 20 to 80°C (Enoki, Shinzato, Sato, Nakamura, & Kamagata, 2011; Hendrickson et al., 2008; Kato, Kosaka, & Watanabe, 2008; Luo, Zhang, Suzuki, Hattori, & Kamagata, 2002; Nazina et al., 2006). Anaerobic digestion systems are operated at temperatures ranging from 20 to 60°C (Ahring, 1995; Ahring, Ibrahim, & Mladenovska, 2001; Ziembinska‐Buczynska, Banach, Bacza, & Pieczykolan, 2014; Zinder et al., 1984). However, quantitative characterization of the cellular details of the response of methanogens to temperature is rare.

*Methanothermobacter thermautotrophicus* delta H is a model thermophilic, hydrogenotrophic, methanogenic *Archaea* (Blaut, 1994; Farhoud et al., 2005; Smith et al., 1997). The optimal temperature for the growth of *Methanothermobacter thermautotrophicus* is 65°C, with the maximum being 75°C and the minimum approximately 40°C. *M. thermautotrophicus* is found widely in natural ecosystems. For example, *M. thermautotrophicus* is highly abundant in thermophilic composting; the methane production potential of mature compost is optimal at 60–65°C. All of the oxygen is rapidly consumed and the thermophilic compost exhibits comparably high production of methane at temperatures above 71°C. Interestingly, *M. thermautotrophicus* is found not only in anaerobic sewage sludge but also in anoxic freshwater sediments. The temperature is usually below 20°C in these anoxic environments, which is well below the observed growth‐temperature range of thermophiles (Kaster et al., 2011). The low temperature below 20°C inhibited their growth, but they survived. Once they returned to a warm temperature, they were able to grow and metabolize again. Our understanding of the genetics and physiology of cold‐adapted methanogens is surprisingly limited, and it is important to evaluate how *M. thermautotrophicus* responds to different temperature stresses, both cold and heat stress, and to investigate its ability to recover from temperature‐stress.

The genome of *M. thermautotrophicus* delta H has been completely sequenced (Smith et al., 1997). Based on this genomic data, investigations are able to obtain much more information about thermophily. However, temperature‐base translational regulation of global proteins expression in *M. thermautotrophicus* under cold shock and high temperature growth stress has not been studied. To better understand the mechanism of this thermophilic methanogen, we used quantitative proteomics to compare the proteomes of *M. thermautotrophicus* delta H before and after exposure to different temperatures. The Isobaric tags for relative and absolute quantitation (iTRAQ) analysis revealed a group of highly up and downregulated genes involved in several major metabolic pathways and membrane functions. This research allowed us to assess the response of *M. thermautotrophicus* to different temperatures and to provide a new level of understanding of methane formation and cellular putative adaptive responses.

## 2 EXPERIMENTAL PROCEDURES

### 2.1 Bacterial growth conditions and high‐ and low‐temperature stress treatments

*Methanothermobacter thermautotrophicus* delta H was obtained from Germany (DSMZ1053). The medium was composed of the following components (per liter of deionized water): MgCl_2_·6H_2_O, 0.1 g; NaCl, 0.6 g; K_2_HPO4, 1.5 g; NH_4_Cl, 1.5 g; sodium cysteine, 0.5 g; NaHCO_3_, 1.0 g; sodium resazurin, 0.001 g; and trace element solutions, 10 ml (MgSO_4_·7H_2_O, 3 g; MnSO_4_·4H_2_O, 0.5 g; NaCl, 10 g; FeSO_4_·7H_2_O, 0.1 g; CoCl_2_·6H_2_O, 0.1 g; CaCl_2_·2H_2_O, 0.1 g; ZnSO_4_·7H_2_O, 0.1 g; CuSO_4_, 0.01 g; KAl(SO_4_)_2_·12H_2_O, 0.01 g; H_3_BO_3_, 0.01 g; Na_2_MoO_4_·2H_2_O, 0.01 g; NiCl_2_·6H_2_O, 0.025 g; and NaSeO_3_·6H_2_O, 0.0003 g). After adjustment of the pH to 7.0, 60 mL of the medium was added to each 600‐mL anaerobic bottle and sterilized under a strictly anaerobic H_2_ and CO_2_ atmosphere (80:20) (Ding, Lv, Zhao, Min, & Yang, 2008; Ding, Peng, Li, Cai, & Yang, 2011; Ding et al., 2010). For high‐temperature treatment, *M. thermautotrophicus* was cultured in this medium at 71°C, and the control was cultured at 65°C using the Hungate technique. For cold shock treatment, *M. thermautotrophicus* was exposed to 4°C for 3 hr after culturing at 65°C in the log phase of growth. The cells at different temperatures were harvested in the log phase of growth at the same time. At this time point, the OD_620_ of the cells is approximately 0.25 for 65°C culture, the OD_620_ of the cells is approximately 0.2 for the 71°C culture. The cells were immediately placed in the plastic centrifuge tubes on ice immediately. Then, the cells were centrifugated at 8,000*g* for 10 min at 4°C.

### 2.2 Protein preparation

The cells were resuspended in the lysis buffer (7 M urea, 2 M thiourea, 4% CHAPS, 40 mM Tris‐HCl (pH 8.5), 1 mM PMSF, and 2 mM EDTA) and sonicated on ice. The protein was reduced with 10 mM DTT (final concentration) at 56°C for 1 hr and then alkylated with 55 mM IAM (final concentration) in a darkroom for 1 hr. The reduced and alkylated protein mixtures were precipitated by adding a 4× volume of chilled acetone at −20°C overnight. After centrifugation at 4°C at 30,000*g*, the pellet was dissolved in 0.5 M TEAE (Applied Biosystems, Milan, Italy) and sonicated on ice. After centrifugation at 30,000*g* at 4°C, an aliquot of the supernatant was extracted for determination of the protein concentration by the Bradford assay. The proteins in the supernatant were stored at −80°C until further analysis (Liu, Chen, Wang, Qiao, & Zhang, 2012; Qiao et al., 2012; Tian, Chen, Wang, Qiao, & Zhang, 2013).

### 2.3 iTRAQ‐LC–MS/MS proteomics analysis

iTRAQ labeling and SCX fractionation, LC‐ESI‐MS/MS analysis based on Q EXACTIVE, and data analysis were performed according to the iTRAQ manufacturer's instructions (Chen et al., 2013; Li et al., 2016; Liu et al., 2012; Qiao et al., 2013, 2012; Tian et al., 2013).

## 3 RESULTS AND DISCUSSION

### 3.1 Functional analysis of the temperature‐dependent proteins

A total of 1,510 unique proteins were identified, representing approximately 83.29% of the 1,813 predicted proteins in the *M. thermautotrophicus* delta H genome (Figure S1, Table S1).

**Figure 1:**
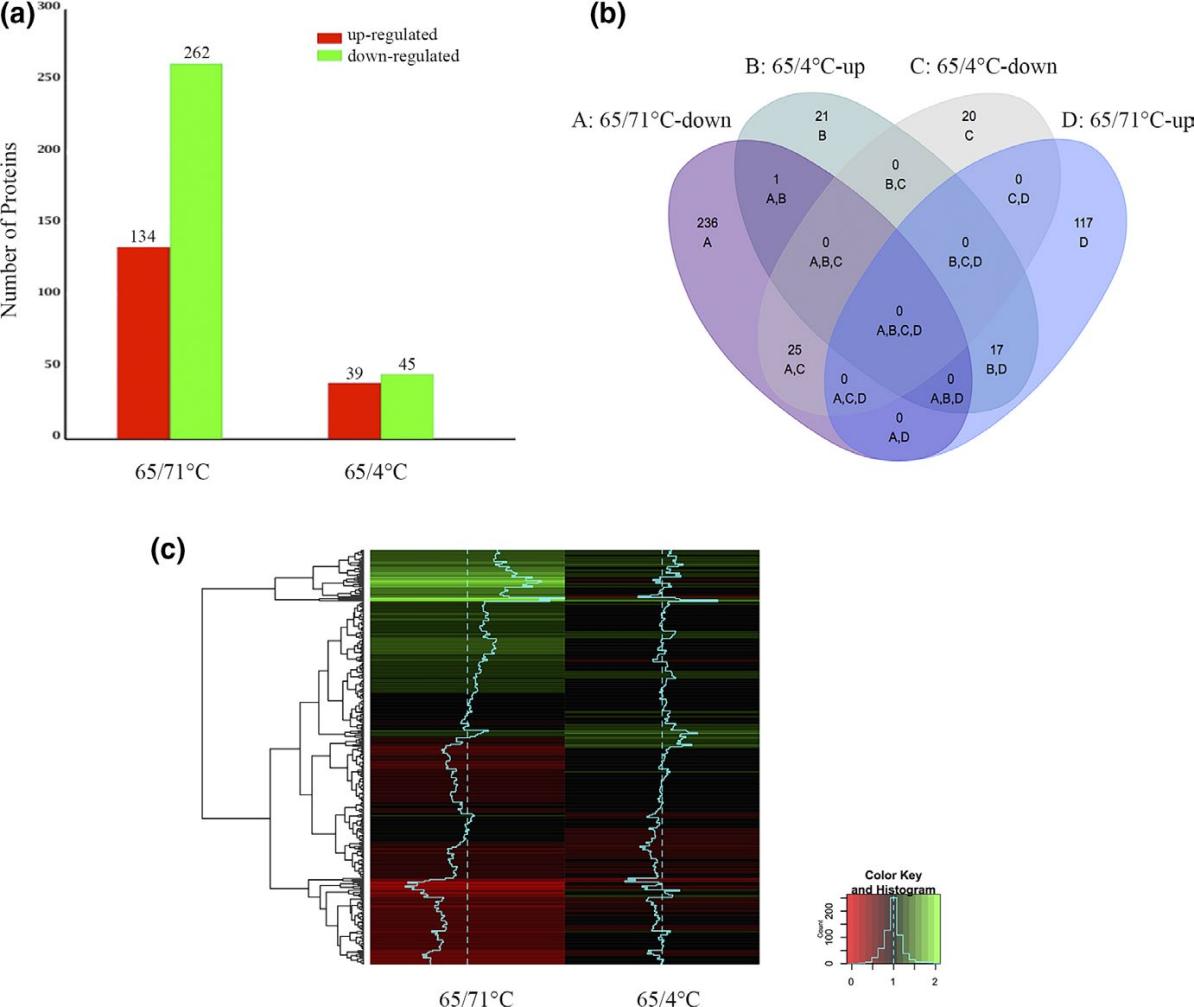
The number of differentially expressed proteins associated with temperature treatment conditions of high temperature growth (71°C) and cold shock (4°C). (a) Number of differentially expressed proteins associated with various temperature‐stress conditions. (b) Venn diagram depicting the overlaps between various temperature‐stress conditions. (c) A heatmap diagram depicting the cluster analysis of the temperature‐dependent proteins

Using a cutoff of 1.2‐fold changes and a *p *< 0.05, we determined that when the culture temperature increased from 65 to 71°C, 134 unique proteins were upregulated and 262 unique proteins were downregulated; when cells were cold shock treated at 4°C for 3 hr, 39 unique proteins were upregulated and 45 unique proteins were downregulated (Figure 1a, Table S2, S3) (Chen et al., 2015; Chu et al., 2015; Hu, Koh, Yoo, Chen, & Wendel, 2015; Mukaihara et al., 2016). Among these proteins, a total of 173 and 307 proteins were upregulated and downregulated, respectively, by the temperature treatments (Figure 1a). Based on Venn analysis, among the differentially expressed proteins, 17 proteins with upregulated expression and 25 with downregulated expression were shared between the high temperature growth (71°C) and cold shock (4°C) treatments; only 1 protein was downregulated at high temperature growth (71°C) and upregulated at cold shock (4°C), while most of the responsive proteins at each of the temperatures were unique based on Venn analysis (Figure 1b). It is possible that there are common mechanisms of biological regulation in *M. thermautotrophicus* delta H at different temperatures; however, most of the responsive proteins were unique for each of the temperature treatments.

**Figure 2:**
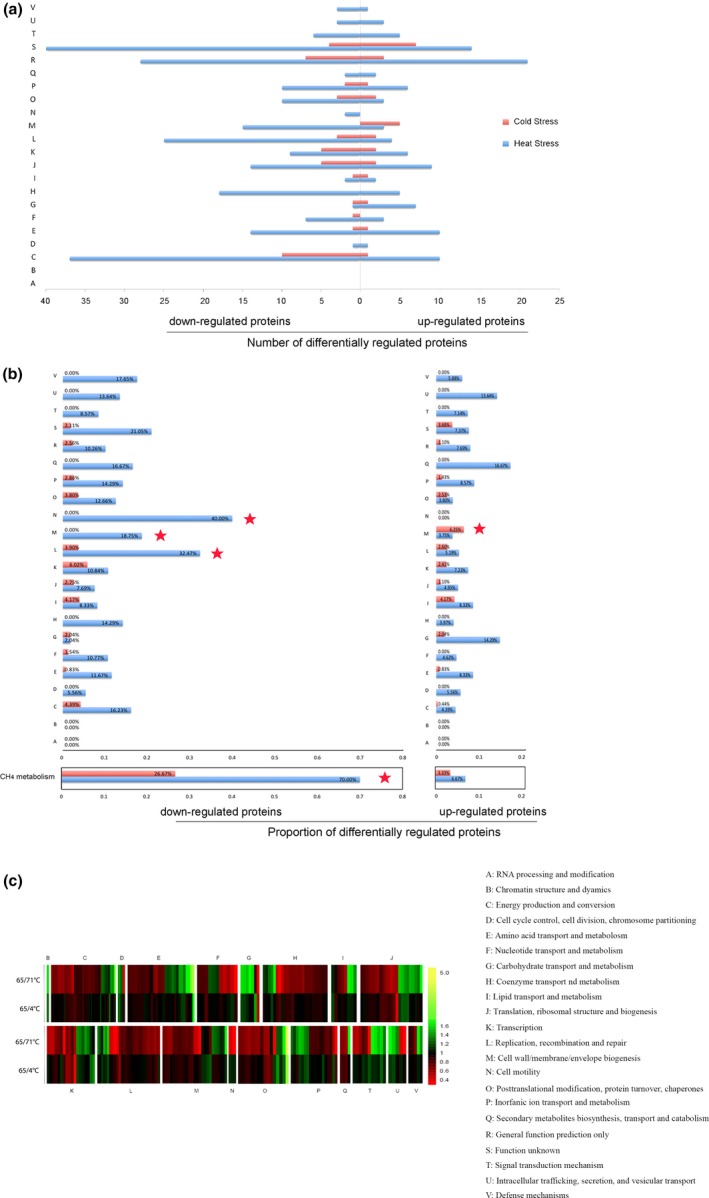
Protein expression patterns of *Methanothermobacter thermautotrophicus* under temperature‐stress. (a) The numbers of significantly up‐ or downregulated (fold change >1.2, *p* < 0.05) proteins in each functional category at high temperature growth (71°C) and cold shock (4°C) are shown. The red histogram indicates all the up‐ and downregulated proteins upon cold shock. The blue histogram indicates all the up‐ and downregulated proteins upon high‐temperature treatment. (b) The proportion of significantly up or downregulated (fold change >1.2, *p* < 0.05) proteins in each functional category at high temperature growth (71°C) and cold shock (4°C) are shown. The red histogram indicates all the up‐ and downregulated proteins upon cold shock. The blue histogram indicates all the up‐ and downregulated proteins upon high‐temperature treatment. (c) A heatmap of the overrepresentation of significantly altered proteins in several biological processes (COG terms)

To understand the biological processes with which the temperature‐dependent proteins are associated, a clusters of orthologous groups (COG) analysis was carried out for the differentially regulated proteins. Based on the COG analysis, we were able to categorize the temperature‐dependent proteins into 22 functional groups (Figure 2). The category in which the largest numbers of proteins were differentially expressed under the temperature‐stress was “energy production and conversion” (Figure 2a). The proportion distributions of the temperature‐dependent proteins were identified as significantly different. Of these 22 COG groups, the proportions of down‐expressed proteins within groups N, L and M were changed 40.00%, 32.47% and 18.75%, respectively, under high‐temperature stress. The proportion within groups C, K, and M were down and upregulated by 4.39%, 6.02%, and 6.25%, respectively, under cold stress, especially the proportion of ‘proteins of methane metabolism’ were downregulated by 70% and 26.67% under both heat‐ and cold stress, respectively (Figure 2b). Notably, except for the ‘cell wall/membrane/envelope biogenesis’ category for the cold shock (4°C) stress and the ‘carbohydrate transport and metabolism’ category for the high temperature growth (71°C) stress, more proteins in each COG category were downregulated than upregulated (Figure 2a).

Previous studies have addressed cold and heat stress in *Bacteria* and *Archaea*. The most prominently upregulated proteins are heat shock proteins, chaperones and other proteins related to protein folding (Campanaro et al., 2011; D'Amico, Collins, Marx, Feller, & Gerday, 2006; Macario, Lange, Ahring, & Conway de Macario, 1999). Many cellular processes, including transcription, translation and membrane fluidity, are regulated (Albers, van de Vossenberg, Driessen, & Konings, 2000; Campanaro et al., 2011; De Maayer, Anderson, Cary, & Cowan, 2014; Wang et al., 2007). It seems that different microbes use different energy metabolism strategies to cope with temperature stress. Some microorganisms reduce energy metabolism under temperature stress, while others increase it (Chen et al., 2013; Goodchild et al., 2004). This finding leads us to hypothesize that *M. thermautotrophicus* delta H downregulates methane metabolic pathways related to energy production and conversion under conditions of temperature stress, and that inhibitory proteins are synthesized as a survival mechanism following temperature stress.

The heatmap (Figure 2c, Table S4) shows the overrepresentation of significantly altered proteins in several biological processes (COG terms). Four functional groups (I, M, P, T) are substantially different between the temperature treatment conditions of high temperature growth (71°C) and cold shock (4°C), which indicates that *M. thermautotrophicus* delta H has different regulatory mechanisms under conditions of high‐ and low‐temperature stress. On the other hand, 5 functional groups (D, C, H, J, L) exhibited similar changes in expression levels in conditions of high‐ and low‐temperature stress, which indicates that these cells have some common compatible mechanisms to cope with conditions of high‐ and low‐temperature stress.

### 3.2 Analysis of the genomic locations of genes coding for the temperature‐dependent proteins

**Figure 3:**
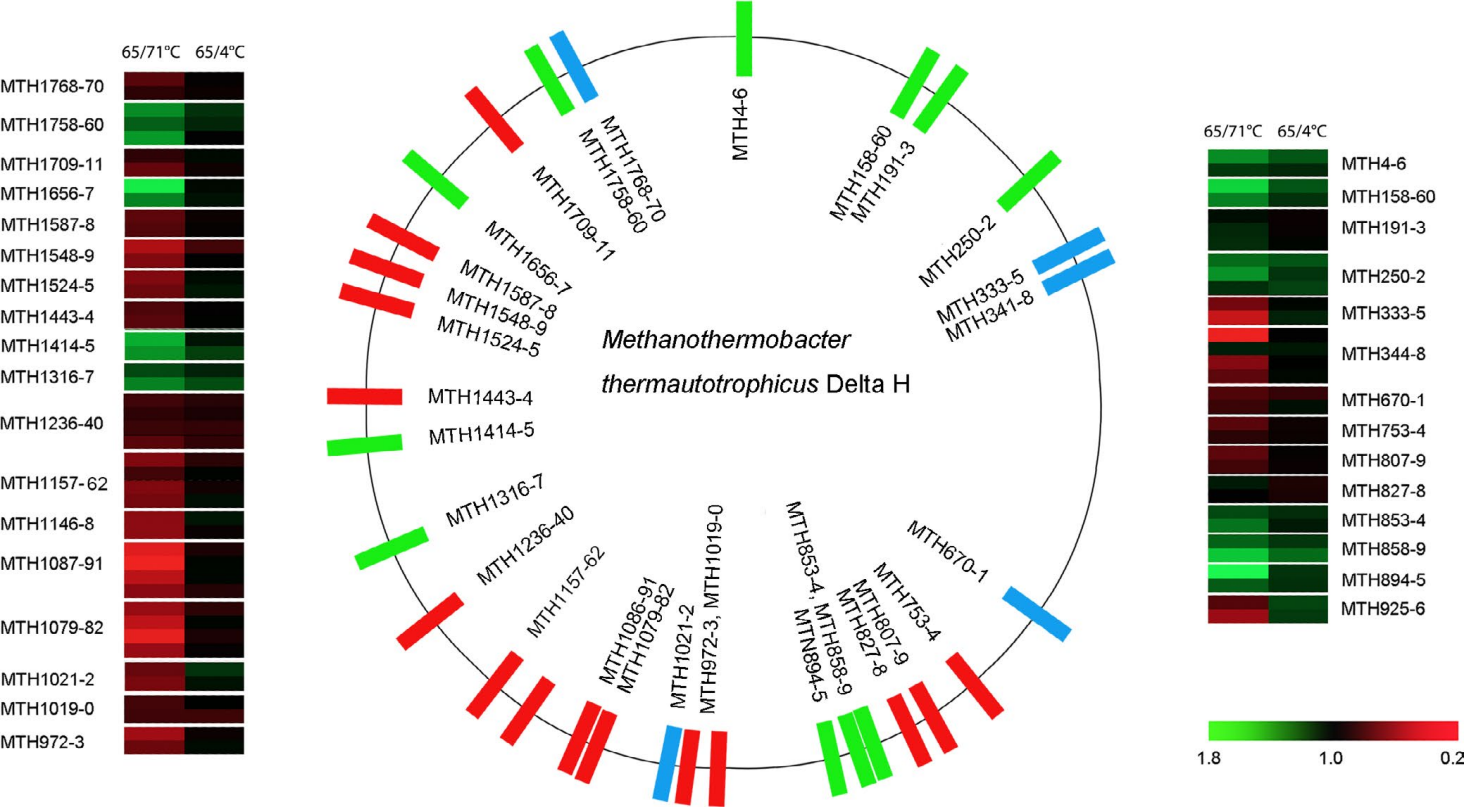
Genomic localization of the gene clusters. The genomic localization of the gene clusters corresponding to the temperature‐dependent proteins that were filtered by cluster analysis. The central panel represents the genomic localization of the temperature‐dependent proteins shown in red (consistently downregulated proteins), blue (variably regulated proteins), and green (consistently upregulated proteins). Right and left panels represent the fold changes in the abundance of each temperature‐dependent protein. The right panel covers the clustered genes from MTH4 to MTH926, and the left panel contains the clustered genes from MTH972 to MTH1770. The lower panel with a color gradient represents the changes in protein abundance from downregulated (red) to upregulated (green) proteins

*Archaea* are typically exposed to an ever‐changing environment in which nutrient availability and abiotic stress may increase or decrease drastically. *Archaea* respond to such variations in their environment by altering their gene expression patterns. Archaeal genes are organized into operons, which are clusters of coregulated genes. Such genetic arrangements allow *Archaea* to rapidly adapt to changes in the environment. To classify the genetic distribution of the temperature‐dependent proteins, we used OperonDB. Some cluster of proteins showing abundance changes in responding to temperature exhibited similar trends (Figure 3, Table S5). Thus, 32 gene clusters covering 77 genes were finally identified. According to the regulation model, the gene clusters were divided into three categories. (1) consistently downregulated proteins, such as MTH1157‐62, all of which were downregulated at both of high temperature growth (71°C) and cold shock (4°C). (2) consistently upregulated proteins, such as MTH4‐6, all of which were upregulated at both high temperature growth (71°C) and cold shock (4°C); and (3) variably regulated proteins, such as MTH344‐8, which showed a mix of up‐ and downregulation at high temperature growth (71°C) and cold shock (4°C).

Intriguingly, most members of these gene clusters belong to the same COG, members of which are expected to perform similar functions. For instance, in the upregulated clusters, MTH4‐6 were annotated as ribosomal proteins. On the other hand, in the downregulated clusters, MTH1157‐62 were annotated as tetrahydromethanopterin S‐methyltransferases, and MTH1548‐9 were annotated as NADH:ubiquinone oxidoreductases. Based on these results, we hypothesize that gene transcription in some clusters is sensitive to temperature, which regulates the activity of the corresponding operon. Thus, similar regulation of proteins encoded in the same predicted operons may exert the thermo‐tolerant functions in *M. thermautotrophicus*. Hence, operon regulation is likely an energy‐efficient method for *M. thermautotrophicus* survival.

### 3.3 Pathway enrichment analysis of the temperature‐responsive proteins

**Figure 4:**
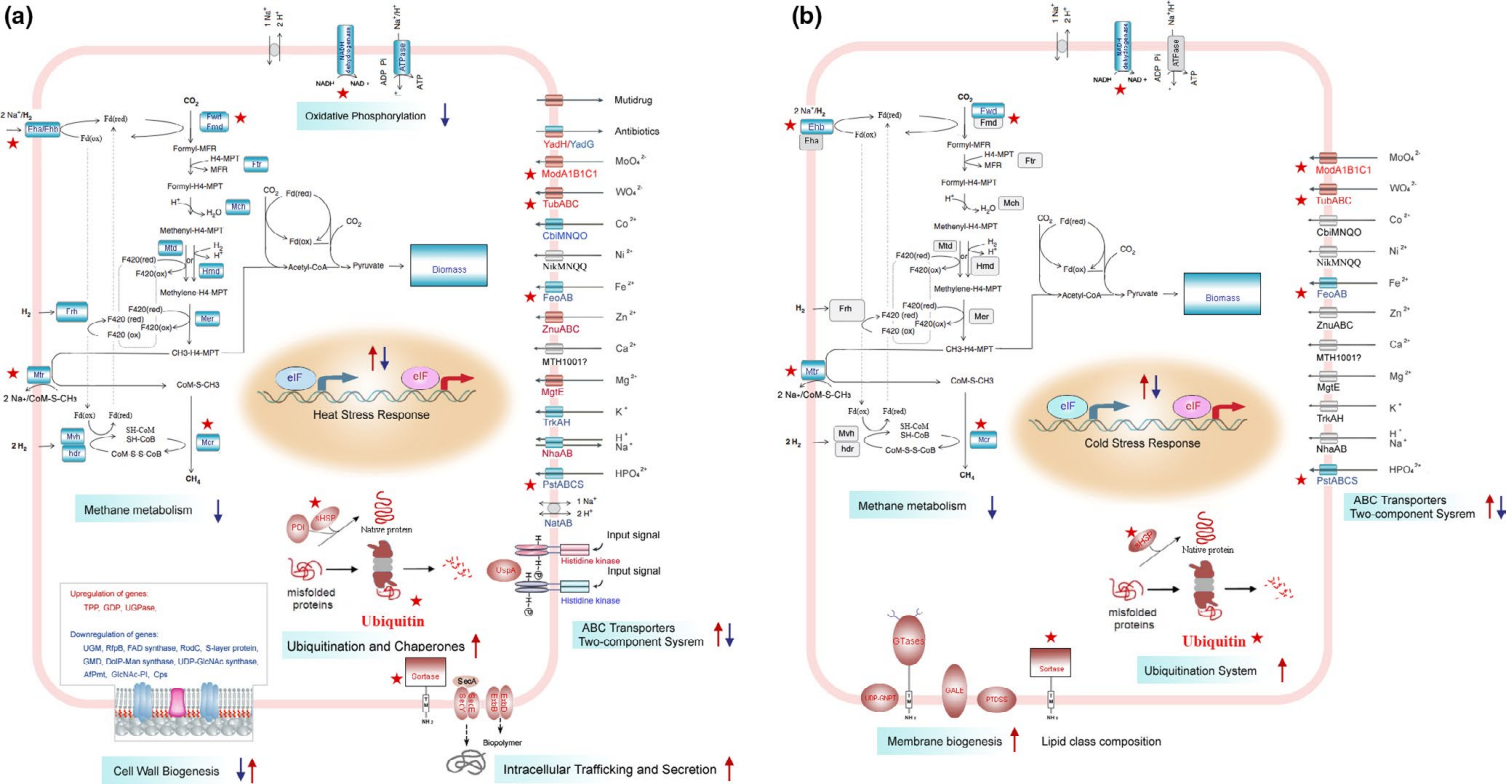
Depiction of *Methanothermobacter thermautotrophicus* proteins involved in methanogenesis and in the survival of the cells that were most strongly affected at high temperature growth (71°C) and cold shock (4°C). The cellular processes most influenced during (a) high temperature growth (71°C) and (b) cold shock (4°C) of the cell. The upregulated and downregulated proteins are highlighted in red and blue boxes, respectively. The relevant upregulated and downregulated KEGG pathways are indicated with red and blue arrows, respectively. Red pentagram indicates: proteins with up‐ or downregulated proteins that were shared between the high temperature growth (71°C) and cold shock (4°C) treatments. KEGG, Kyoto encyclopedia of genes and genomes

To identify the cellular metabolic pathways affected by temperature, metabolic pathway enrichment analysis was carried out for the differentially regulated proteins to identify the cellular metabolic pathways affected by temperature. The responsive proteins were matched to the proteins annotated by using the Kyoto encyclopedia of genes and genomes (KEGG) pathway database, and then the frequencies of the responsive proteins of different temperature‐stress treatments were then compared with each KEGG pathway. Based on the evidence above, we propose a model mechanism for methane formation and survival of *M. thermautotrophicus* (Table S6, Figure 4).

### 3.4 Proteins involved in CH_4_ formation from CO_2_ and H_2_ and in energy conservation

“Methane metabolism” (ko00680) is a core energy‐generation process. *M. thermautotrophicus* is a species of *Archaea* representative of hydrogenotrophic methanogens, which generate energy by using H_2_ to reduce CO_2_ to methane. The pathway model for methane formation from CO_2_ and H_2_ and energy metabolism in *M. thermautotrophicus* is depicted in Figure 4. In the pathway, many proteins are involved in methane formation from H_2_ and CO_2_ and in energy conservation, including tungsten formyl‐methanofuran dehydrogenase, F_420_‐nonreducing [NiFe]‐hydrogenase, heterodisulfide reductase, F_420_‐reducing hydrogenase, hydrogen‐dependent methylene‐H_4_MPT dehydrogenase, formyl‐methanofuran dehydrogenase, formylmethanofuran‐H_4_MPT N‐formyltransferase, methenyl‐H_4_MPT cyclohydrolase, coenzyme F_420_‐dependent methylene‐H_4_MPT dehydrogenase, F_420_‐dependent N_5_N_10_‐methylene‐H_4_MPT reductase, N_5_‐methyl‐H_4_MPT:HS‐CoM methyltransferase, Methyl‐CoM reductase (Fwd, Mvh, hdr, Frh, Hmd, Fmd, Ftr, Mch, Mtd, Mer, Mtr, Mcr) (Kaster et al., 2011; Luo et al., 2002). Notablely, most of these proteins were downregulated at both high temperature growth (71°C) and cold shock (4°C) (Figure 4). *M. thermautotrophicus* produces methane as the major metabolic end product of a unique energy‐generation process. Our proteomic data supports the hypothesis that energy generation by *M. thermautotrophicus* was decreased in order to maintain low biomass. The effects of temperature stress on growth and/or viability can account for the much slower growth rates at high temperature growth (71°C) and cold shock (4°C).

“Oxidative phosphorylation” (ko00190) is involved in electron transfer and energy‐generation. To generate energy, electrons are transferred to membrane‐bound oxidoreductase systems by the cofactors F_420_ and ferredoxin, and the resulting electron flow generates a proton gradient that drives ATP synthesis and allows NADPH to be regenerated (Williams et al., 2011). Archaeal NADH dehydrogenase and ATPase were present at low levels at 71°C, and NADH dehydrogenase was also present at low levels at 4°C, which confirmed that the low level of energy‐generation in *M. thermautotrophicus* is involved in the high‐ and low‐temperature stress responses.

### 3.5 Environmental information processing

Proteins in the category “environmental information processing”, including those categorized as “ABC transporters” (ko02010), “two‐component system” (ko02020) and “bacterial secretion system” (ko03070), were differentially regulated by different temperatures. ABC transporters play a wide variety of physiological roles; the functions of ABC transporters encompass detoxification, nutrient uptake, iron channels, stress adaptation, and export of cellular components (Albers et al., 2000; Higgins, 2001; Jungwirth & Kuchler, 2006). Two‐component systems have been recognized as critical stimulus–response mechanisms; the stimulus is sensed by a histidine kinase (HK) and transmitted to a response regulator (Bhate, Molnar, Goulian, & DeGrado, 2015; Dintner et al., 2011; Heermann & Jung, 2010; Vidova, Bobalova, & Smigan, 2011). Together with neighboring ABC transporter systems, two‐component systems form specific stress–response modules in *M. thermautotrophicus*. Detailed analysis showed that proteins involved in ion transport were differentially regulated at high and low temperatures (Figure 4). Bacteria secrete proteins via various secretion systems to adapt to their growth environments. The protein translocase subunit SecY/E and the biopolymer transport protein ExbB/D are important secretory‐system proteins. Both SecY/E and ExbB/D were upregulated at 71°C. The differential protein expression levels of “environmental information processing” proteins are caused by the transfer of signals from the environment to networks of signal transduction pathways, resulting in the regulation of gene expression (Figure 4).

### 3.6 Protein folding and degradation

Many proteins involved in protein folding and degradation were significantly upregulated, including protein disulfide isomerase (PDI), which is essential for the formation and cleavage of disulfide bonds and for protein folding; small heat shock protein (sHSP), which aids with proper protein folding; and the ubiquitin protein, which is associated with the degradation of misfolded proteins. One striking feature of the proteome was that the ubiquitin protein was especially abundant (upregulated 5.12‐fold at high temperature growth (71°C) and 2.23‐fold at cold shock (4°C)). High and low temperatures are likely to denature proteins, and the correct folding of the nascent polypeptides was likely to be attenuated. Furthermore, the high levels of unfolded and aggregated proteins resulting from high‐temperature stress damage led to these proteins being frequently targeted for protein turnover.

### 3.7 Replication and repair

“Replication and repair” proteins, including those annotated as “homologous recombination” (ko03440), “nucleotide excision repair” (ko03420), “base excision repair” (ko03410), “DNA replication” (ko03030) and “mismatch repair” (ko03430), were differently regulated by different temperatures. DNA and RNA helicases, which are involved in homologous recombination; excinuclease ABC subunit ACD (Uvr ACD), which is involved in nucleotide excision repair and mismatch repair; endonuclease III, which is involved in base excision repair; and DNA primase, which is involved in DNA replication were all downregulated at both high temperature growth (71°C) and cold shock (4°C). However, proteins involved in DNA degradation, such as exonuclease III and DNase, were upregulated at both high temperature growth (71°C) and cold shock (4°C). The analysis allows us to speculate that *M. thermautotrophicus* tends to directly initiate protein‐ or DNA‐degradation processes instead of repair mechanisms in response to temperature stress.

### 3.8 Cell walls and cell membranes response

Archaeal cell walls and cell membranes respond rapidly to temperature stress. Modulation of cell wall and cell membrane structure caused by temperature stress resulted in pronounced changes in the organization of the cell walls and cell membranes (Albers et al., 2000; Campanaro et al., 2011; De Maayer et al., 2014; Wang et al., 2007). Our proteomic analysis showed that exposure to high temperatures induced rapid regulation of proteins involved in lipopolysaccharide biosynthesis (ko00540) (1.68%), glycosphingolipid biosynthesis (ko00601) (0.67%), pseudopeptidoglycan biosynthesis (ko00550) (0.67%), and glycerolipid metabolism (ko00561) (1.01%) and in the biosynthesis of unsaturated fatty acids (ko01040) (0.34%). Exposure to low temperatures induces rapid regulation of proteins involved in glycerolipid metabolism (ko00561) (1.85%) and in the biosynthesis of unsaturated fatty acids (ko01040) (1.85%) (Table S6). In particular, the upregulation of sortase, UGPase, TPP, and GDP facilitated adaptation to high‐temperature growth (71°C) stress by enhancing the wall stability, changing the membrane fluidity and changing the lipid and protein composition of the membrane. In contrast, the expression of genes encoding other cell wall and cell membrane proteins and structures, such as UGM, RfpB, FAD synthase, RodC, AfPmt, s‐layer protein, Cps, UDP‐GlcNAc synthase, GDM, DolP‐Man synthase, and GlcNAc‐PI, was generally suppressed at 71°C. Therefore, modulation of cell wall and cell membrane structures plays an essential role in the response to condition of high‐temperature growth stress. Based on these results, we monitored the cell wall morphology using SEM. *M. thermautotrophicus* cells cultured at 65°C presented long, irregularly curved, fully formed rods. The cell morphologies was severely deformed when the cells were cultured at 71°C (Figure 4).

Many of the putative cell wall/membrane/envelope proteins, such as UDP‐GNPT, sortase, GALE and PTDSS, exhibited higher abundances under cold shock at 4°C. This observation indicates that processes promoting cold response occur at the cell envelope/wall/membrane (De Maayer et al., 2014; Goodchild et al., 2004).

### 3.9 Concluding remarks

This study used the iTRAQ technique to provide a global view of protein regulation in *M. thermautotrophicus* exposed to conditions of high‐ and low‐temperature stress. We identified the differential expression of proteins required for the synthesis of the enzymes, coenzymes, and prosthetic groups involved in the energy metabolism of CO_2_ reduction to methane.

Based on these findings, we propose that while there are common mechanisms of biological regulation in *M. thermautotrophicus* under different temperature conditions, most of the responsive proteins are unique for each of the temperatures (Dong & Chen, 2012; Li et al., 2015). Notably, when exposed to both high‐ and low‐temperature stress, *M. thermautotrophicus* decreased CH_4_‐metabolism to maintain low biomass, and the proteins involved in protein folding and degradation were significantly upregulated. Some proteins annotated as “ABC transporters”, “two‐component system”, “bacterial secretion system”, and “cell membrane/wall/envelope biogenesis” were differentially regulated by high‐ and low‐temperature stress (Figure 2, 4).

**Figure 5:**
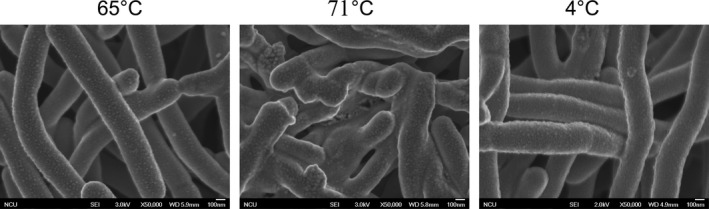
The SEM images of *Methanothermobacter thermautotrophicus* in different temperature treatment. The cellular morphology and ultrastructure was influenced during high temperature growth (71°C) and cold shock (4°C) states of the cell. Bar indicates 100 nm

One of the distinctive characteristics of *M. thermautotrophicus* is the presence of a complex membrane system (Zeikus & Wolfe, 1973). The morphology and ultrastructure of *M. thermautotrophicus* change greatly at both low and high growth temperatures (Figure 5). We found that “cell membrane/wall/envelope biogenesis” constituted the largest proportion (6.25%) of proteins upregulated under cold shock (4°C), while 18.75% of the proteins downregulated under high‐temperature growth stress (71°C) belonged to the “cell membrane/wall/envelope biogenesis” groups (Figure 2a). Our data demonstrate that both of high temperatures growth and cold shock can induce the significant different expression of proteins involved in “cell membrane/wall/envelope biogenesis” (Figures 3, 4).

One somewhat unanticipated finding was that the ubiquitin protein was especially abundant under conditions of both high‐ and low‐temperature stress. High and low temperatures are likely to denature proteins, and high levels of unfolded and aggregated proteins resulting from temperature stress damage lead to these proteins being generally targeted for degradation if they cannot be effectively refolded to the correct protein structures (Maupin‐Furlow, 2013). These findings suggest that *M. thermautotrophicus* has complex mechanisms of translation regulation, which facilitate the survival of this organism in unstable ecosystems with fluctuating temperatures (Campanaro et al., 2011).

Low‐temperature environments (≤5°C) cover approximately 70‐80% of the earth's surface. Low temperature effects all components and processes within cells (Murray & Grzymski, 2007). Only some cold‐adapted (psychrophilic) microorganisms possess specific characteristics that promote their growth at low temperature (Cavicchioli, 2006). By contrast, their growth of cold‐shock (mesophilic and thermophilic) microorganisms were inhibited at the low temperature, but they can survive. It is interesting to understand the different global gene expression patterns between cold‐shock and cold‐adapted microorganisms. A variety of global proteomic analyses have been performed focusing on growth at 4°C (Burg et al., 2010; Goodchild, Raftery, Saunders, Guilhaus, & Cavicchioli, 2005; Nichols et al., 2004; Ting et al., 2010). Response to low temperature is characterized by upregulation of genes for cell surface proteins, tRNA modification, and specific RNA‐binding proteins, ribosomal proteins and proteins involved in secretion (Campanaro et al., 2011; Goodchild et al., 2005). In our study, low temperature shock was characterized by upregulation of proteins for cell wall/membrane/envelope biogenesis, lipid transport and metabolism, along with downregulation of proteins for transcription, energy production and conversion (Figure 2).

It is noteworthy that the higher growth temperature (71°C) is an organism with a multi‐days adjusted growth rate while the colder shock (4°C) treatment is a few hours (3 hr) unlikely to be a growth adjusted cultured in the experiment. The short‐term cold shock treatment may led to no significant difference. For example, there is a major difference in downregulated methane metabolism proteins were 70% of proteins in the higher growth temperature (71°C), and only ~27% in the cold shock (4°C). However, if that cold condition was maintained for some days is likely that a significantly higher fraction will be downregulated too. Therefore, the roles of altered short‐term and long term growth rates at suboptimal temperatures need to be distinguished with caution.

Overall, our study has provided a comprehensive understanding of methane production by *M. thermautotrophicus* and of the cellular responses of this organism to high‐ temperature growth and low‐temperature shock stress.

## AVAILABILITY OF DATA AND MATERIALS

The mass spectrometric proteomic data have been deposited to the ProteomeXchange Consortium via the PRIDE (Perez‐Riverol et al., 2016; Vizcaino et al., 2016) partner repository with the dataset identifier PXD006685. Reviewer account details: Username: reviewer51531@ebi.ac.uk; Password: oq0efHAr.

## AUTHOR CONTRIBUTIONS

Cong Liu and Lihui Mao have contributed equally to this work.

## CONFLICT OF INTEREST

No conflict of inerest.
