# Supplementary figures and images for: Comparative proteomic analysis of Methanothermobacter thermautotrophicus reveals methane formation from H2 and CO 2 under different temperature conditions
Source: Microbiologyopen. 2018 Sep 10;8(5):e00715. doi: 10.1002/mbo3.715 (PMC6528648; doi:10.1002/mbo3.715)

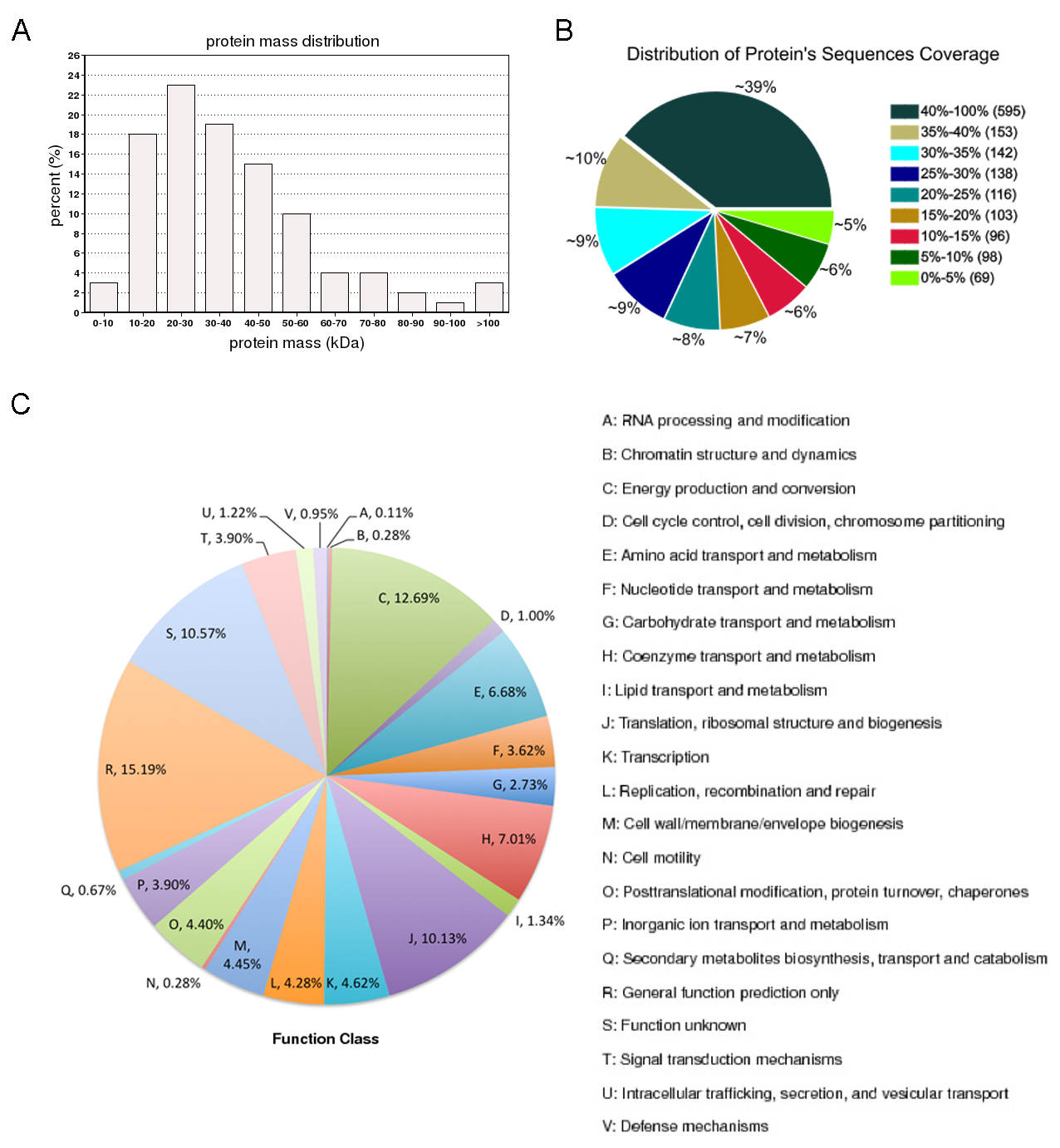

Supplement: Supplementary file 1 [file MBO3-8-e00715-s001.tif]

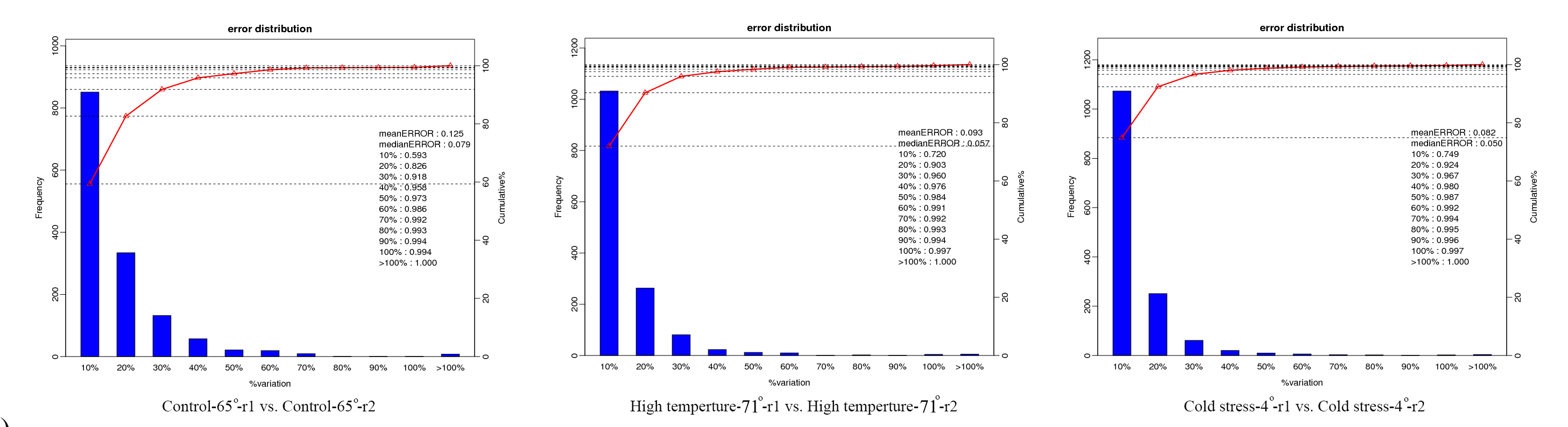

Supplement: Supplementary file 2 [file MBO3-8-e00715-s002.tif]
